# Supplementary material for: Immunogenicity and Safety of Omicron-Containing Multivalent COVID-19 Vaccines in Unvaccinated and Previously Vaccinated Adults
Source: Vaccines (Basel). 2024 Sep 27;12(10):1109. doi: 10.3390/vaccines12101109 (PMC11510771; doi:10.3390/vaccines12101109)
Supplement: Supplementary file 1 [file vaccines-12-01109-s001.zip › vaccines-3185570-supplementary.pdf]

Supplementary Materials 2
Table S1: Seroresponse rate of neutralizing antibodies against BA.5 in Cohort 1. 2
Table S2: Seroresponse rate of neutralizing antibodies against BA.5 in Cohort 2. 2
Table S3: Safety analysis set in Cohort 1. 3
Table S4: Safety analysis set in Cohort 2. 4
Figure S1: GMTs of neutralizing antibodies against BA.5 in the negative N-protein antibody group. 6
Figure S2: GMTs of neutralizing antibodies against BA.5 in the positive N-protein antibody group. 6

Supplementary Materials

Supplementary Table S1: Seroresponse rate of neutralizing antibodies against BA.5 in Cohort 1.

	SCTV01E (N = 62)	SCTV01E-1 (N = 55)
Omicron BA.5		
Day 42		
SRR n/N	88.7 (55/62)	89.1 (49/55)
95% CI of SRR	78.1, 95.3	77.8, 95.9
Rate Difference (95% CI) SCTV01E-1 vs. SCTV01E		1.0 (-10.6, 12.7)
P value		0.8594
Day 150		
SRR n/N	82.1 (32/39)	77.8 (28/36)
95% CI of SRR	66.5, 92.5	60.8, 89.9
Rate Difference (95% CI) SCTV01E-1 vs. SCTV01E		0.8 (-16.6, 18.1)
P value		0.9322
Day 178		
SRR n/N	87.2 (34/39)	83.3 (30/36)
95% CI of SRR	72.6, 95.7	67.2, 93.6
Rate Difference (95% CI) SCTV01E-1 vs. SCTV01E		-0.3 (-16.4, 15.9)
P value		0.9744

SRR, Seroresponse Rate; CI, Confidence Interval.

Supplementary Table S2: Seroresponse rate of neutralizing antibodies against BA.5 in Cohort 2.

	SCTV01E (N = 112)	SCTV01E-1 (N = 112)
Omicron BA.5		
Day 28		
SRR n/N	79.5 (89/112)	79.5 (89/112)
95% CI of SRR	70.8, 86.5	70.8, 86.5
Rate Difference (95% CI) SCTV01E-1 vs. SCTV01E		-0.1 (-10.7, 10.6)
P value		0.9921
Day 120		
SRR n/N	67.4 (60/89)	62.4 (58/93)
95% CI of SRR	56.7, 77.0	51.7, 72.2
Rate Difference (95% CI) SCTV01E-1 vs. SCTV01E		-5.8 (-19.7, 8.0)
P value		0.4121
Day 148		
SRR n/N	75.0 (63/84)	76.9 (70/91)
95% CI of SRR	64.4, 83.8	66.9, 85.1
Rate Difference (95% CI) SCTV01E-1 vs. SCTV01E		2.3 (-10.5, 15.1)
P value		0.7237

SRR, Seroresponse Rate; CI, Confidence Interval.

Supplementary Table S3: Safety analysis set in Cohort 1.

	SCTV01E (N = 79) n (%)	SCTV01E-1 (N = 81) n (%)
**Any TEAEs**	10 (12.7)	11 (13.6)
Within 15min after vaccination	0	0
Within 7 days after vaccination	6 (7.6)	10 (12.3)
Within 28 days after vaccination	10 (12.7)	11 (13.6)
**Any Investigational Vaccine Related TEAEs**	8 (10.1)	10 (12.3)
Within 15min after vaccination	0	0
Within 7 days after vaccination	6 (7.6)	10 (12.3)
Within 28 days after vaccination	8 (10.1)	10 (12.3)
**Any ≥ Grade 3 and above TEAEs**	1 (1.3)	1 (1.2)
Any ≥ Grade 3 investigational vaccine related TEAEs	1 (1.3)	1 (1.2)
**Solicited TEAEs**	6 (7.6)	5 (6.2)
Any investigational vaccine-related solicited TEAEs	6 (7.6)	5 (6.2)
**Unsolicited TEAEs**	8 (10.1)	9 (11.1)
Any investigational vaccine unsolicited TEAEs	6 (7.6)	8 (9.9)
**SAEs**	0	0
Investigational vaccine-related SAEs	0	0
**AESI**	0	0
Investigational vaccine-related AESI	0	0

TEAE, Treatment Emergent Adverse Event; SAE, Serious Adverse Event; AESI, Adverse Event of Special Interest.

Supplementary Table S4: Safety analysis set in Cohort 2.

	SCTV01E (N = 119) n (%)	SCTV01E-1 (N = 121) n (%)
**Any TEAEs**	24 (20.2)	33 (27.3)
Within 15 min after vaccination	0	0
Within 7 days after vaccination	22 (18.5)	29 (24.0)
Within 28 days after vaccination	24 (20.2)	32 (26.4)
**Any Investigational Vaccine-related TEAEs**	23 (19.3)	31 (25.6)
Within 15 min after vaccination	0	0
Within 7 days after vaccination	22 (18.5)	29 (24.0)
Within 28 days after vaccination	23 (19.3)	31 (25.6)
**Any ≥ Grade 3 TEAEs**	2 (1.7)	3 (2.5)
Any ≥ grade 3 and above investigational vaccine-related TEAEs	2 (1.7)	2 (1.7)
**Solicited TEAEs**	13 (10.9)	1 (13.2)
Any investigational vaccine solicited TEAEs	13 (10.9)	16 (13.2)
**Unsolicited TEAEs**	13 (10.9)	21 (17.4)
Any investigational vaccine unsolicited TEAEs	12 (10.1)	19 (15.7)
**SAEs**	0	2 (1.7)
Investigational vaccine-related SAEs	0	1 (0.8)
**AESI**	0	1 (0.8)
Investigational vaccine-related AESI	0	1 (0.8)

TEAE, Treatment Emergent Adverse Event; SAE, Serious Adverse Event; AESI, Adverse Event of Special Interest.

Supplementary Table S5: Acronyms/ Abbreviations List

SCTV01C	Bivalent protein vaccine [Alpha/Beta]
SCTV01E	Tetravalent protein vaccine [Alpha/Beta/Delta/Omicron (BA.1)]
SCTV01E-1	Tetravalent protein vaccine [Alpha/Beta/Delta/Omicron (BA.1/BA.4/5)]
SCT-VA02B	Adjuvant
CHO	Chinese Hamster Ovary
IgG	Immunoglobulin G
NMPA	National Medicinal Product Agency
GMT	Geometric Mean Titers
GMR	Geometric Mean Ratios
GMFR	Geometric Mean Fold Rises
LSGMT	Least Square Geometric Mean Titers
nAb	Neutralizing Antibody

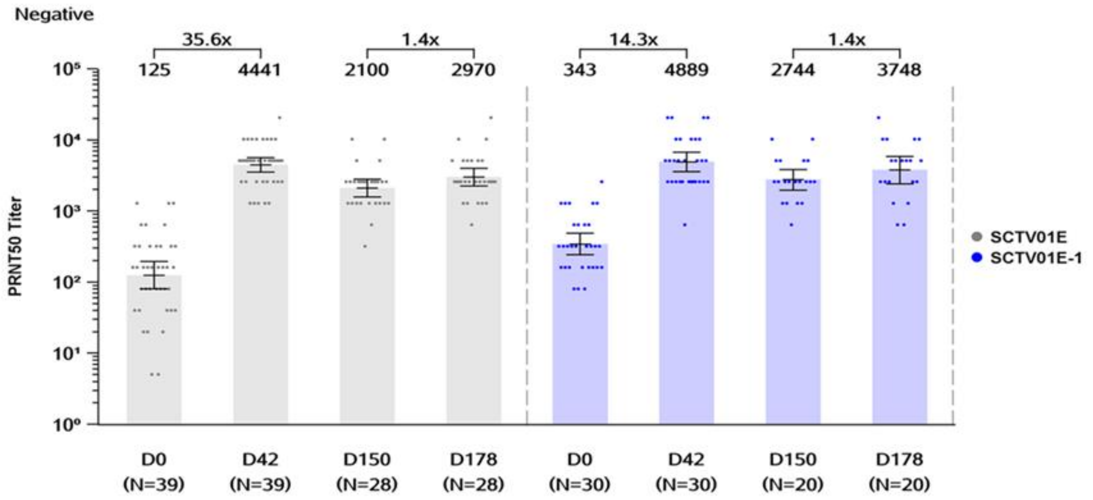

Supplementary Figure S1: GMTs of neutralizing antibodies against BA.5 in the negative N-protein antibody group.

GMTs of neutralizing antibody were measured using a 50% plaque reduction neutralization test (PRNT50). Bars show the GMTs on days 0, 28,120 and 148. Center of the bars represents the GMT. Dots represent the values of r individual participants. Note: SCTV01E group (grey), SCTV01E-1 group (blue). Abbreviations: GMT, geometric mean titer; PRNT50, 50% plaque reduction neutralization test

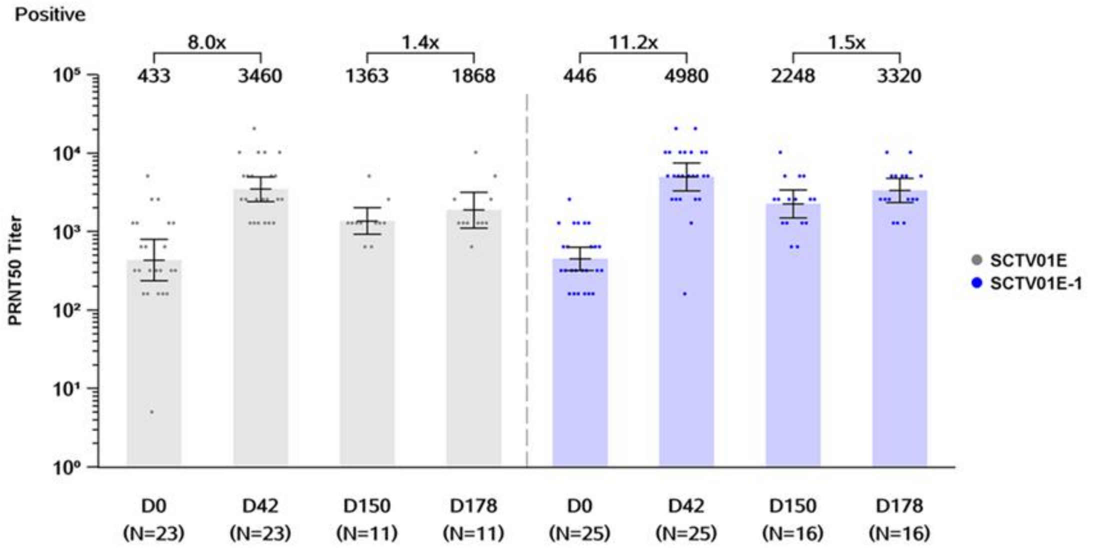

Supplementary Figure S2: GMTs of neutralizing antibodies against BA.5 in the positive N-protein antibody group.

GMTs of neutralizing antibody were measured using a 50% plaque reduction neutralization test (PRNT50). Bars show the GMTs on days 0, 28,120 and 148. Center of the bars represents the GMT. Dots represent the values of individual participants. Note: SCTV01E group (grey), SCTV01E-1 group (blue). Abbreviations: GMT, geometric mean titer; PRNT50, 50% plaque reduction neutralization test
